# The function of *Mef2c* toward the development of excitatory and inhibitory cortical neurons

**DOI:** 10.3389/fncel.2024.1465821

**Published:** 2024-09-23

**Authors:** Claire Ward, Lucas Sjulson, Renata Batista-Brito

**Affiliations:** ^1^Dominick P. Purpura Department of Neuroscience, Albert Einstein College of Medicine, Bronx, NY, United States; ^2^Department of Psychiatry and Behavioral Sciences, Albert Einstein College of Medicine, Bronx, NY, United States; ^3^Department of Genetics, Albert Einstein College of Medicine, Bronx, NY, United States

**Keywords:** cortical development, MEF2C haploinsufficiency syndrome, autism spectrum disorder, GABA, interneurons

## Abstract

Neurodevelopmental disorders (NDDs) are caused by abnormal brain development, leading to altered brain function and affecting cognition, learning, self-control, memory, and emotion. NDDs are often demarcated as discrete entities for diagnosis, but empirical evidence indicates that NDDs share a great deal of overlap, including genetics, core symptoms, and biomarkers. Many NDDs also share a primary sensitive period for disease, specifically the last trimester of pregnancy in humans, which corresponds to the neonatal period in mice. This period is notable for cortical circuit assembly, suggesting that deficits in the establishment of brain connectivity are likely a leading cause of brain dysfunction across different NDDs. Regulators of gene programs that underlie neurodevelopment represent a point of convergence for NDDs. Here, we review how the transcription factor MEF2C, a risk factor for various NDDs, impacts cortical development. Cortical activity requires a precise balance of various types of excitatory and inhibitory neuron types. We use MEF2C loss-of-function as a study case to illustrate how brain dysfunction and altered behavior may derive from the dysfunction of specific cortical circuits at specific developmental times.

## Introduction

Neurodevelopmental disorders (NDDs), such as autism spectrum disorder (ASD), intellectual disability, and schizophrenia, represent a leading cause of neuropsychiatric illness, affecting one in eight children in the US (Bitta et al., 2017). We remain far from understanding the complex biology underlying NDDs; however, recent human genetic studies have consistently shown large degrees of genetic overlap between distinct NDDs (International Schizophrenia Consortium et al., 2009; Williams et al., 2011; Cross-Disorder Group of the Psychiatric Genomics Consortium, 2013; Insel and Landis, 2013; Ruderfer et al., 2014; Cross-Disorder Group of the Psychiatric Genomics Consortium, 2019). Recent work shows that subtle genetic changes, especially during critical developmental periods, can cause diverse impairments in brain activity and behavior. NDD risk-associated genes are highly expressed during neonatal development, a sensitive period for NDDs notable for cortical circuit assembly and synaptic maturation (LeBlanc and Fagiolini, 2011; Willsey et al., 2013; Meredith, 2015; Marin, 2016; Satterstrom et al., 2020). These findings have introduced the possibility that changes to a small set of common signaling pathways might result in various NDD phenotypes. The identification of these common signaling pathways is a fundamental aim of NDD research. Consistent with this idea, NDDs often present with a shared set of core symptoms and feature common biomarkers, such as changes in brain synchrony and cortical oscillations (Bartos et al., 2007; Milh et al., 2007; Lakatos et al., 2008; Allene and Cossart, 2010; Tan et al., 2013; Fryer et al., 2016; Hwang et al., 2016; Seibt et al., 2016).

Using bioinformatics and ever-growing genetic data, many known NDD-associated genes have been classified according to cellular and circuit functions. Numerous cellular processes are impaired across NDDs, including transcriptional and epigenetic regulation, neuronal proliferation, neuronal migration, neuronal survival, connectivity of both excitatory and inhibitory circuits (Rossignol, 2011; Meredith, 2015; Marin, 2016; Krol and Feng, 2018), and synaptic transmission (Chattopadhyaya and Cristo, 2012; Clement et al., 2012; Meredith et al., 2012; Estes et al., 2015; Meredith, 2015; Canitano and Pallagrosi, 2017; Krol and Feng, 2018; Parenti et al., 2020). Transcription factors, a group of proteins that interact with DNA and regulate gene expression through modulation of RNA synthesis, represent a major point of convergence for neurodevelopmental and neuropsychiatric diseases (Santos-Terra et al., 2021). One of such transcription factors is the myocyte enhancement factor 2c protein (MEF2C), which is encoded by the MEF2C gene in humans and the orthologous Mef2c gene in mice. MEF2C has been widely associated with various NDDs such as autism spectrum disorder (ASD), schizophrenia, bipolar disorder (Harrington et al., 2016; Rajkovich et al., 2017; Tu et al., 2017; Assali et al., 2019). In this review we will treat *Mef2c* as a test case for how changes in one gene can impact excitatory and inhibitory cortical circuits, and will focus on how dysfunction of *Mef2c* in different cortical circuits relates to pathological pattens of activity and altered behavior.

## *Mef2c* and neurodevelopmental disorders

**FIGURE 1:**
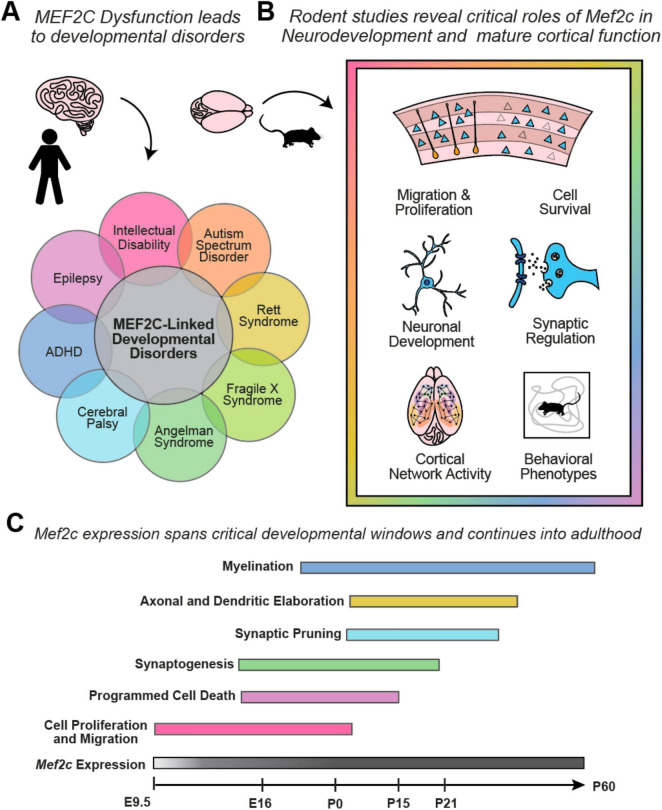
**(A)** Dysfunction of the *MEF2C* gene in humans can give rise to many neurodevelopmental disorders, which often are characterized by overlapping behavioral phenotypes. **(B)** Transgenic mouse studies of the *Mef2c* gene have identified MEF2C as a transcription factor that regulates gene programs that are critical to many cellular processes. Loss of *Mef2c* expression can give rise to impaired neuronal activity across cell populations and leads to behavioral phenotypes typical of mouse models of neurodevelopmental disorders, such as hyperactivity in an open field arena. **(C)** *Mef2c* is expressed from early embryonic development through adulthood, with roles in shaping gene expression during critical neurodevelopmental windows and regulating synapses in mature cortical circuits.

Human genome-wide association studies (GWAS) reveal that *MEF2C* is a common genetic risk factor for various neurodevelopmental and neuropsychiatric disorders (Gandal et al., 2018; Harrington et al., 2020; Fahey et al., 2023). Microdeletions or coding-region missense or nonsense mutations in *MEF2C* during development can lead to *MEF2C* haploinsufficiency syndrome, which often features intellectual disability, epilepsy, and autism spectrum disorder (Borlot et al., 2019; Chaudhary et al., 2021; Cooley Coleman et al., 2021; Cooley Coleman et al., 2022), and loss of *Mef2c* in rodent models leads to profound changes in behaviors associated with NDDs (Barbosa et al., 2008; Li H. et al., 2008; Adachi et al., 2016; Harrington et al., 2016; Deczkowska et al., 2017; Tu et al., 2017; Bjorness et al., 2020; Harrington et al., 2020; Li et al., 2023; Cho et al., 2024; Ward et al., 2024) (Figure 1A).

The symptom severity associated with MEF2C loss-of-function, along with the number of NDDs associated with it, underscore its necessity in regulating genetic programs during neurodevelopment. Gene ontology studies in mice revealed that *Mef2c* regulates processes such as neurogenesis, neuronal differentiation and morphogenesis, cell survival, and synapse development (Harrington et al., 2016; Tu et al., 2017; Harrington et al., 2020; Allaway et al., 2021). Indeed, *Mef2c* knockdown mice exhibit many cellular deficits, such as reduced neurogenesis, increased cell death, and increased excitatory to inhibitory (E/I) neurotransmission (Tu et al., 2017) (Figure 1B). In addition to the many cellular phenotypes, *Mef2c* knockdown mice exhibit behavioral phenotypes common to mouse models of NDDs, such as hyperactivity and deficits in social interactions (Tu et al., 2017; Harrington et al., 2020; Li et al., 2023), enabling us to investigate the circuits involved in *Mef2c*-related pathophysiology and behavior.

## *Mef2c* regulates the cellular and synaptic development of excitatory and inhibitory neurons

*Mef2c* is highly expressed within the cerebral cortex throughout the life of an animal, including during embryonic development. *Mef2c* is broadly expressed in pyramidal cells, the principal excitatory cells of the cortex, and subsets of inhibitory interneurons (Assali et al., 2019). Within GABAergic cortical interneurons *Mef2c* is expressed in virtually all parvalbumin-expressing INs (PV-INs) and a subset of somatostatin expressing INs (SST-INs) (Mayer et al., 2018; Ward et al., 2024). Using conditional genetics, and with the increasing availability of mouse Cre-driver lines that can be used to target specific cell types across various stages of development, we have gained a granularity that allows for better understanding of how *Mef2c*-mediated gene regulation is critical for the development of excitatory and inhibitory circuits, as detailed in Table 1.

**TABLE 1:** Cellular and behavioral phenotypes in *Mef2c* mouse models.

Models			
*Mef2c*+/− (Haploinsufficiency model)		Single copy from all cells, embryonic	Tu et al., 2017; Harrington et al., 2020; Li et al., 2023
Nestin-Cre:*Mef2c*^F/null^ or *Mef2c*^F/F^		Neuronal progenitor, embryonic	Li H. et al., 2008; Chen et al., 2016
GFAP-Cre:*Mef2c*^F/F^ (GFAP^cKO^)		Radial glial cells, embryonic	Barbosa et al., 2008
Emx1-Cre:*Mef2c*^F/F^ (Emx1^cKO^)		Excitatory forebrain neurons, embryonic	Harrington et al., 2016
Emx1-Cre:*Mef2c*^F/+^ (Emx1^cHet^)		“”	Harrington et al., 2020
CAMKII-Cre:*Mef2c*^F/F^ (CAMKII^cKO^)		Excitatory forebrain neurons, postnatal	Adachi et al., 2016
Ascl-CreER:*Mef2c*^F/F^ (Aslc^cKO^)		Hippocampal adult born neurons	Basu et al., 2024
VGAT-Cre:*Mef2c*^F/+^ (VGAT^cHet^)		GABAergic neurons, embryonic	Cho et al., 2024
Lhx6-Cre:*Mef2c*^F/F^ (Lhx6^cKO^)		PV-IN and SST-IN progenitors, embryonic	Ward et al., 2024
Lhx6-Cre:*Mef2c*^F/+^(Lhx6^cHet^)		“”	Ward et al., 2024
PV-Cre:*Mef2c*^F/F^ (PV^cKO^)		PV-INs, postnatal	Ward et al., 2024
PV-Cre:*Mef2c*^F/+^ (PV^cHet^)		“”	Harrington et al., 2020
SST-Cre:*Mef2c*^F/+^ (SST^cHet^)		SST-INs, embryonic	Cho et al., 2024
VIP-Cre:*Mef2c*^F/+^ (VIP^cHet^)		VIP-INs, embryonic	Cho et al., 2024
Cx3Cr1-Cre^ER^:*Mef2c*^F/+^ (Cx3Cr1^cHet^)		Microglia, embryonic	Deczkowska et al., 2017; Harrington et al., 2020
Cx3Cr1-Cre^ER^:*Mef2c*^F/+^ (Cx3Cr1^cHet^)		Microglia, embryonic	Harrington et al., 2020
**Cellular phenotypes**			
Dendritic Spines		= *Mef2c*^+/–^	Harrington et al., 2020
		↓ *Mef2c*^+/–^	Li et al., 2023
		↑ GFAP^cKO^	Barbosa et al., 2008
		↓ Emx1^cKO^	Harrington et al., 2016
		↑ CAMKII^cKO^	Adachi et al., 2016
		↓ Aslc^cKO^	Basu et al., 2024
sEPSC or mEPSC amplitude and frequency		↓ Amp ↑ Freq *Mef2c*^+/–^	Tu et al., 2017
		↓ Amp ↑ Freq *Mef2c*^+/–^ (Layer 2/3 cell)	Harrington et al., 2020
		↓ Amp = Freq *Mef2c*^+/–^ (Layer 5 cell)	Harrington et al., 2020
		= Amp ↓Freq *Mef2c*^+/–^	Li et al., 2023
		↓ Amp ↓ Freq Nestin-Cre:*Mef2c*^F/null^	Li H. et al., 2008
		= Amp ↑ Freq GFAP^cKO^	Barbosa et al., 2008
		↓ Amp ↓ Freq Emx1^cKO^	Harrington et al., 2016
		↓ Amp ↓ Freq Emx1^cHet^	Harrington et al., 2020
		= Amp ↓ Freq Aslc^cKO^	Basu et al., 2024
		= Amp = Freq VGAT^cHet^	Cho et al., 2024
		= Amp ↓ Freq Lhx6^cKO^	Ward et al., 2024
		= Amp ↓ Freq Lhx6^cHet^	Ward et al., 2024
		= Amp = Freq PV^cKO^	Ward et al., 2024
		= Amp ↓ Freq Cx3Cr1^cHet^	Harrington et al., 2020
Inhibitory cells and synaptic markers		↓ PV-INs *Mef2c*^+/–^	Tu et al., 2017
		↓ PV-INs *Mef2c*^+/–^	Li et al., 2023
		= PV-INs VGAT^cHet^	Cho et al., 2024
		↓ PV-INs Lhx6^cKO^	Ward et al., 2024
		↓ PV-INs Lhx6^cHet^	Ward et al., 2024
		= PV-INs PV^cKO^	Ward et al., 2024
		↓ GAD65 *Mef2c*^+/–^	Tu et al., 2017
		↑ GAD65 Emx1^cKO^	Harrington et al., 2016
		↓ VGAT *Mef2c*^+/–^	Tu et al., 2017
sIPSC or mIPSC amplitude and frequency		↓ Amp ↓ Freq *Mef2c*^+/–^	Tu et al., 2017
		= Amp = Freq *Mef2c*^+/–^	Harrington et al., 2020
		= Amp = Freq *Mef2c*^+/–^	Li et al., 2023
		↑ Amp ↑ Freq Emx1^cKO^	Harrington et al., 2016
		= Amp = Freq Aslc^cKO^	Basu et al., 2024
		= Amp = Freq VGAT^cHet^	Cho et al., 2024
		= Amp ↓ Freq Lhx6^cKO^	Ward et al., 2024
		= Amp ↓ Freq Lhx6^cHet^	Ward et al., 2024
**Behavioral phenotypes**			
Hyperactivity	Open field track length or beam breaks	= *Mef2c*^+/–^	Tu et al., 2017
		↑ *Mef2c*^+/–^	Harrington et al., 2020
		↑ *Mef2c*^+/–^	Li et al., 2023
		= Nestin-Cre:*Mef2c*^F/null^	Li H. et al., 2008
		= GFAP^cKO^	Barbosa et al., 2008
		↑ Emx1^cKO^	Harrington et al., 2016
		↑ Emx1^cHet^	Harrington et al., 2020
		↑ CAMKII^cKO^	Adachi et al., 2016
		= Aslc^cKO^	Basu et al., 2024
		= VGAT^cHet^	Cho et al., 2024
		↑ Lhx6^cKO^	Ward et al., 2024
		= Lhx6^cHet^	Ward et al., 2024
		= PV^cKO^	Ward et al., 2024
		= SST^cHet^	Cho et al., 2024
		= VIP^cHet^	Cho et al., 2024
		= Cx3Cr1^cKO^	Deczkowska et al., 2017
		= Cx3Cr1^cHet^	Harrington et al., 2020
Hyperactivity	Jumping	↑ *Mef2c*^+/–^	Harrington et al., 2020
		↑Emx1^cKO^	Harrington et al., 2016
		↑ Emx1^cHet^	Harrington et al., 2020
		= VGAT^cHet^	Cho et al., 2024
		↑ Lhx6^cKO^	Ward et al., 2024
		= Lhx6^cHet^	Ward et al., 2024
		= PV^cKO^	Ward et al., 2024
		= PV^cHet^	Harrington et al., 2020
		= SST^cHet^	Cho et al., 2024
		↑ VIP^cHet^—Males only	Cho et al., 2024
		↑ Cx3Cr1^cHet^—Males only	Harrington et al., 2020
Motor	Paw clasping	↑ *Mef2c*^+/–^	Tu et al., 2017
		↑ Nestin-Cre:*Mef2c*^F/null^	Li H. et al., 2008
		↑ GFAP^cKO^	Barbosa et al., 2008
		↑ CAMKII^cKO^	Adachi et al., 2016
		↑ Lhx6^cKO^	Ward et al., 2024
		= Lhx6^cHet^	Ward et al., 2024
		= PV^cKO^	Ward et al., 2024
Motor	Ex. Rotarod, balance beam	= *Mef2c*^+/–^	Tu et al., 2017
		= *Mef2c*^+/–^	Harrington et al., 2020
		↓ Emx1^cKO^	Harrington et al., 2016
		↓ CAMKII^cKO^	Adachi et al., 2016
Social	3-Chamber social preference Time spent with social stimuli	↓ *Mef2c*^+/–^	Tu et al., 2017
		↓ *Mef2c*^+/–^	Harrington et al., 2020
		= *Mef2c*^+/–^	Li et al., 2023
		↓ Emx1^cKO^	Harrington et al., 2016
		= Emx1^cHet^	Harrington et al., 2020
		= CAMKII^cKO^	Adachi et al., 2016
		= Aslc^cKO^	Basu et al., 2024
		↓ VGAT^cHet^—Females only	Cho et al., 2024
		↓ Lhx6^cKO^	Ward et al., 2024
		= Lhx6^cHet^	Ward et al., 2024
		= PV^cKO^	Ward et al., 2024
		= PV^cHet^	Harrington et al., 2020
		= SST^cHet^	Cho et al., 2024
		= VIP^cHet^	Cho et al., 2024
		↓ Cx3Cr1^cHet^	Harrington et al., 2020
		↓ Cx3Cr1^cKO^	Deczkowska et al., 2017
Social	Ultrasonic vocalizations	↓ *Mef2c*^+/–^	Harrington et al., 2020
		↓ Emx1^cKO^	Harrington et al., 2016
		↓ Nestin^cKO^	Chen et al., 2016
		= VGAT^cHet^	Cho et al., 2024
Exploration	Elevated plus maze Open arm time or track length	↑ *Mef2c*^+/–^	Harrington et al., 2020
		↑ Nestin-Cre:*Mef2c*^F/null^	Li H. et al., 2008
		= GFAP^cKO^	Barbosa et al., 2008
		↑ Emx1^cHet^	Harrington et al., 2020
		= PV^cHet^	Harrington et al., 2020
		↑ VGAT^cHet^—Females only	Cho et al., 2024
		↑ Lhx6^cKO^	Ward et al., 2024
		= Lhx6^cHet^	Ward et al., 2024
		= SST^cHet^	Cho et al., 2024
		= VIP^cHet^	Cho et al., 2024
Cognitive	Barnes maze	↑ *Mef2c*^+/–^	Tu et al., 2017
	Escape latency	= *Mef2c*^+/–^	Harrington et al., 2020
Cognitive	Fear conditioning time spent freezing	= *Mef2c*^+/–^	Harrington et al., 2020
		↑ Nestin-Cre:*Mef2c*^F/null^	Li H. et al., 2008
		↓ GFAP^cKO^	Barbosa et al., 2008
		↓ Emx1^cKO^	Harrington et al., 2016
		↓ Aslc^cKO^	Basu et al., 2024
		= VGAT^cHet^	Cho et al., 2024
		↑ SST^cHet^—Females only	Cho et al., 2024
		= VIP^cHet^	Cho et al., 2024

Comparison of transgenic mouse models of *Mef2c* loss-of-function, with an emphasis on studies containing cellular characterizations within the cortex. *Mef2c* Haploinsufficiency models feature a disrupted expression of a single *Mef2c* allele in all cells, while conditional loss-of-function models are cell-type specific, with varied Cre-dependent removal times and numbers of affected alleles. Select cellular and behavioral characterizations common across mouse models are compared. No change in phenotype relative to wildtype animals is denoted as “=,” while “↑” and “↓” indicate increases or decreases in a given cellular or behavioral phenotype, respectively.

### Neurogenesis, neuronal differentiation, and survival

The MEF2C transcription factor is critical during development and regulates neurogenesis, neuronal differentiation, and survival (Mao et al., 1999; Li H. et al., 2008; Li Z. et al., 2008) (Figure 1C). Removal of the *Mef2c* gene in Nestin-expressing neural stem/progenitor cells impairs neuronal proliferation and results in disrupted layer formation at embryonic day 18.5 (E18.5) and disorganized cortical plate at postnatal day 7 (P7) (Li H. et al., 2008). Deficits in neuronal migration may be a common feature in ASDs, and despite the distinct progenitor pools and migratory streams of excitatory and inhibitory neurons, deficits in *Mef2c* expression have been shown to affect both cell types (Li H. et al., 2008; Reiner et al., 2016). This effect appears to be dependent on factors such as cell type, dose, and developmental stage, as we highlight across many molecular and behavioral phenotypes in this review. Embryonic removal of *Mef2c* from excitatory neurons alone does not lead to gross changes in cortical structure, while overexpression of *Mef2c* in adult born neurons leads to mislocalization in the hippocampus (Harrington et al., 2016; Basu et al., 2024). MEF2C continues to be expressed in postmitotic pyramidal cells, where it promotes their survival by activating anti-apoptotic genes and inhibiting pro-apoptotic pathways, thus highlighting its protective role in the nervous system (Li Z. et al., 2008; Tu et al., 2017). MEF2C promotes differentiation by regulating the expression of genes involved in neuronal differentiation and maturation, such as *Bdnf* (Lyons et al., 2012; Harrington et al., 2016; Tu et al., 2017; Avarlaid et al., 2024), and has been shown to regulate excitatory neurons’ dendritic arborization, and axonal guidance (Ma and Telese, 2015; Harrington et al., 2016; Tu et al., 2017; Basu et al., 2024). Embryonic overexpression of *Mef2c* increases basal dendritic arborization (Jung et al., 2016), while reduced *Mef2c* results in decreased arborization (Tu et al., 2017). MEF2C has been shown to positively modulate the expression of MECP2 (Zweier et al., 2010; Paciorkowski et al., 2013), a transcriptional regulator that facilitates expression of the KCC2 ion channel that is critical for developmental transition from the excitatory to inhibitory of action GABA (Tang et al., 2016; Oyarzabal et al., 2020), and it is possible that this aspect of cellular development could be affected by *Mef2c* loss of function as well. In addition, depletion of MEF2C leads to the defective activation of the mTOR pathway (Pereira et al., 2009) and mTOR positively regulates MEF2C (Wu et al., 2015). Furthermore, members of the MEF2C interactome are strongly linked to mTOR pathway activity, such as MECP2 (Ricciardi et al., 2011; Tsujimura et al., 2015; Rangasamy et al., 2016; Olson et al., 2018; Zhang et al., 2018) and FMR1 (Sharma et al., 2010; Casingal et al., 2020; Zhang et al., 2024). These serve as examples of the many gene regulatory networks implicated in neurodevelopmental disorders that are upstream or downstream of *Mef2c* (D’Haene et al., 2019; Zhang and Zhao, 2022). *Mef2c* is also expressed in cortical interneurons originating in the medial ganglionic eminence (MGE) (Mayer et al., 2018; Ward et al., 2024). *Mef2c* is expressed in cortical parvalbumin interneuron (PV-IN) precursors during embryonic development, where it functions as the earliest indicator of PV-IN fate and is necessary for the survival and molecular maturation of the PV-IN lineage (Mayer et al., 2018). *Mef2c* continues to be expressed in PV-INs throughout the animal’s life, but postnatal removal of *Mef2c* using the PV^Cre^ mouse line does not impact PV-IN survival or differentiation (Ward et al., 2024). *Mef2c* is also expressed by roughly 30% of SST-INs, however, its loss does not impact SST-IN survival or differentiation (Ward et al., 2024).

### Synapse development and function

*Mef2c* regulates synaptic transmission and plays a critical role in synapse development and plasticity of cortical pyramidal cells (Barbosa et al., 2008; Li H. et al., 2008; Adachi et al., 2016; Harrington et al., 2016; Rajkovich et al., 2017; Tu et al., 2017; Harrington et al., 2020; Basu et al., 2024). *Mef2c* influences the expression of synaptic proteins, such as Arc and SynGAP, which are involved in synaptic strength and plasticity (Flavell et al., 2006; Harrington et al., 2016; Tu et al., 2017; Puang et al., 2020); however, the role of *Mef2c* in synaptic development is complex. Selective loss of *Mef2c* in cortical pyramidal cells can result in either an increased (Barbosa et al., 2008; Adachi et al., 2016) or decreased (Harrington et al., 2016; Tu et al., 2017; Li et al., 2023) density of dendritic spines, with various changes in electrophysiological synaptic properties (Barbosa et al., 2008; Li Z. et al., 2008; Adachi et al., 2016; Harrington et al., 2016; Tu et al., 2017; Harrington et al., 2020; Ward et al., 2024). These seemingly opposing roles underscore the nuanced role of *Mef2c* in synapse maturation and/or maintenance, which is input- (local versus long-range) and activity-dependent (Rajkovich et al., 2017). *Mef2c* strengthens long-range inputs from the contralateral cortex and weakens local inputs (Rajkovich et al., 2017). Interestingly, *Mef2c* only results in weakened synaptic strength in the presence of sensory experience, highlighting the role of activity in *Mef2c-*dependent function. In addition to the role of sensory experience on *Mef2c* function, *Mef2c* activity is also dependent on sleep (Bjorness et al., 2020). Sleep deprivation leads to *Mef2c*-dependent upregulation of synapse-weakening genes and a reduction in synapse-strengthening genes, as well as changes in glutamatergic synaptic transmission and sleep-dependent synaptic remodeling (Bjorness et al., 2020). *Mef2c* is also necessary for the synaptic maturation of PV-INs (Ward et al., 2024). Embryonic loss of *Mef2c* in MGE INs (Lhx6-Cre:Mef2c^F/F^) causes a massive reduction in glutamatergic synaptic transmission onto INs, but postnatal loss of *Mef2c* by using the line PV^Cre^ does not impact synaptic transmission onto PV-INs (Ward et al., 2024), thus highlighting the critical role of *Mef2c* during development.

### E/I imbalance

Imbalances in excitatory and inhibitory synaptic transmission, particularly increased E/I balance, have been proposed to cause various NDDs, including ASD and schizophrenia (Rubenstein and Merzenich, 2003; Gao and Penzes, 2015; Sohal and Rubenstein, 2019; Liu et al., 2021). The E/I imbalance hypothesis was originally based on the observation that patients with NDDs have increased rates of epilepsy, which is true for *MEF2C* haploinsufficiency patients (Cooley Coleman et al., 2021; Wan et al., 2021; Cooley Coleman et al., 2022). However, GABA_*A*_ agonists or positive allosteric modulators, which reduce E/I balance, do not alleviate the core symptoms of NDDs (Lingjaerde, 1991), and conditions that increase E/I balance, such as withdrawal from alcohol or sedative/hypnotics (Hendricson et al., 2007), do not cause syndromes resembling NDDs, calling this hypothesis into question. Increasingly, the field is moving away from the overly simplistic one-dimensional E/I imbalance hypothesis to more sophisticated models of cortical circuit dysfunction (O’Donnell et al., 2017; Sohal and Rubenstein, 2019) that incorporate multiple distinct excitatory and inhibitory cell types. The complex cell-type-dependent and temporally-specific effects of *MEF2C* haploinsufficiency provide a compelling illustration of why this more nuanced approach is necessary.

## Impact of *Mef2c* on behavior

Despite their complex etiologies, NDDs feature overlapping behavioral traits. Most *Mef2c* loss-of-function mouse models include assays for behavioral domains affected in various NDDs (Table 1). While there is no set battery of tests conducted across NDD studies, there are several phenotypes that stand out across studies. Loss or reduction of *Mef2c* in both pyramidal cells and MGE interneurons leads to hyperactivity (Adachi et al., 2016; Harrington et al., 2016; Harrington et al., 2020; Li et al., 2023; Cho et al., 2024; Ward et al., 2024), paw clasping (Barbosa et al., 2008; Li H. et al., 2008; Adachi et al., 2016; Tu et al., 2017; Ward et al., 2024), and social deficits (Harrington et al., 2016; Deczkowska et al., 2017; Tu et al., 2017; Harrington et al., 2020; Li et al., 2023; Basu et al., 2024; Cho et al., 2024; Ward et al., 2024). *Mef2c* haploinsufficiency mice (*Mef2c*^+/–^) exhibited hyperactivity and social deficit phenotypes; however, not all assays were in agreement (Tu et al., 2017; Harrington et al., 2020; Li et al., 2023). One study reported that *Mef2c*^+/–^ animals exhibit impaired spatial memory in the Barnes maze and Morris water maze (Tu et al., 2017), however, a separate study of *Mef2c*^+/–^ animals revealed deficits in a battery of cognitive tests including fear conditioning, Barnes maze, y-maze, and an operant reward learning test (Harrington et al., 2020), thus illustrating the challenge comparing behavioral domains across studies.

An advantage of mouse models and the advent of conditional genetics is the ability to investigate how specific brain circuits contribute to pathological neuronal activity and altered behavior (see behavior phenotypes resulting from reduced *Mef2c* in various cell types in Table 1). For instance, Harrington and colleagues observed that both *Mef2c*^+/–^ animals and animals with embryonic loss of *Mef2c* specifically in pyramidal cells (Emx1-Cre:*Mef2c*^F /F^ animals) exhibit a significant reduction in the frequency of ultrasonic vocalizations in pups, with adult animals emitting fewer and less complex vocalizations (Harrington et al., 2016; Harrington et al., 2020). On the other hand, Emx1-Cre:*Mef2c*^F /F^ animals exhibited cognitive deficits in contextual memory fear conditioning while none were observed in *Mef2c*^+/–^ animals when tested on the same paradigm (Harrington et al., 2016; Harrington et al., 2020). Another important factor in considering how the loss of a gene impacts behavior is the age at which the loss occurred. For instance, early (embryonic) but not late (postnatal) removal of *Mef2c* from MGE interneurons leads to hyperactivity (Ward et al., 2024). Furthermore, some studies have found that some behavioral phenotypes are sex specific (Harrington et al., 2020; Cho et al., 2024). There is evidence to suggest that anxiety-like behavior correlates with *Mef2c* expression in a way that is influenced by sex and estrous cycle phase (Jaric et al., 2019; Hong et al., 2024), further underscoring the importance of considering sex as a biological variable. These findings provide an example of how relating gene dysfunction to behavior is extremely challenging; however, investment in relating changes in brain activity with target behaviors holds the promise of revealing the circuits underlying neurodevelopmental behaviors in NDDs.

## Discussion

Brain development is a pivotal and meticulously orchestrated process, governed by numerous neurobiological pathways that lay the foundation for essential brain functions. It is likely that various NDDs share pathways and circuits that become altered during prenatal or early postnatal stages. A main challenge in NDD treatment is the fact that diagnosis often occurs after the most effective time for treatment. Early identification of biomarkers for NDDs is critical given its potential for early intervention. Another big challenge is that despite significant advancements in understanding the pathophysiology of NDDs, targeted and effective treatments are still rare. Identification of common NDD molecular pathways and circuit dysfunction could potentially serve as an early prognostic indicator for NDDs and allow for targeted therapies.

The convergence of genetic factors and the existence of critical periods of vulnerability for NDDs underscores the potential for drugs to target fundamental networks as soon as possible in order to prevent or mitigate clinical manifestations of NDDs. However, more work needs to be done to establish a causal link between molecular and circuit abnormalities, disease pathology, and abnormal behavior. Animal models offer the opportunity to comprehensively unravel the molecular, circuit, and temporal intricacies of NDDs, pinpointing potential therapeutic targets, and ultimately informing us about new treatment modalities. In particular, the mouse as a disease model can provide valuable tools in deciphering the intricate genetic landscape of NDDs as well as elucidating the molecular, cellular, and circuit impacts of diverse mutations toward brain development and disease physiology. Interestingly, pharmacological and gene therapy approaches have both been applied in *Mef2c*^+/–^ models, rescuing both cellular and behavioral phenotypes (Tu et al., 2017; Li et al., 2023). Nonetheless, for animal models to be useful it is important to consider the fundamental differences between animal models and humans, especially during development.

Single-cell omics techniques, alongside non-invasive brain activity measures like EEGs, computational models, and bioinformatics network analysis, offer a pathway to delineate parallels between animal models and humans. These methodologies have the potential to unravel the intricate relationship between gene expression alterations during human brain neuronal development and the manifestation of behaviors associated with NDDs. This understanding holds promise for developing tailored therapeutic interventions, catalyzing a transition from the prevailing symptom-based approach toward more proactive, targeted, and effective treatments.
